# Association between *MAPT* haplotype and memory function in patients with Parkinson's disease and healthy aging individuals

**DOI:** 10.1016/j.neurobiolaging.2014.12.006

**Published:** 2015-03

**Authors:** Sophie E. Winder-Rhodes, Adam Hampshire, James B. Rowe, Jonathan E. Peelle, Trevor W. Robbins, Adrian M. Owen, Roger A. Barker

**Affiliations:** a Department of Clinical Neurosciences, University of Cambridge, Cambridge, UK; b Department of Child and Adolescent Psychiatry, Institute of Psychiatry, King's College London, UK; c MRC Cognition and Brain Sciences Unit, Cambridge, UK; d The Division of Brain Sciences, Department of Medicine, Imperial College, London, UK; e Behavioural and Clinical Neuroscience Institute, University of Cambridge, Cambridge, UK; f Department of Otolaryngology, Washington University in St. Louis, St. Louis, MO, USA; g The Brain and Mind Institute, The University of Western Ontario, London Ontario, Canada

**Keywords:** Parkinson's disease, Dementia, Aging, Memory, fMRI, Hippocampus, *MAPT*, Genetics, Tau, Cognitive impairment, Picture recognition

## Abstract

Genetic variation is associated with differences in the function of the brain as well as its susceptibility to disease. The common H1 haplotypic variant of the microtubule-associated protein tau gene (*MAPT*) has been related to an increased risk for Parkinson's disease (PD). Furthermore, among PD patients, H1 homozygotes have an accelerated progression to dementia. We investigated the neurocognitive correlates of *MAPT* haplotypes using functional magnetic resonance imaging. Thirty-seven nondemented patients with PD (19 H1/H1, 18 H2 carriers) and 40 age-matched controls (21 H1/H1, 19 H2 carriers) were scanned during performance of a picture memory encoding task. Behaviorally, H1 homozygosity was associated with impaired picture recognition memory in PD patients and control subjects. These impairments in the H1 homozygotes were accompanied by an altered blood-oxygen level-dependent response in the medial temporal lobe during successful memory encoding. Additional age-related differences in blood-oxygen level-dependent response were observed in the medial temporal lobes of H1 homozygotes with PD. These results suggest that common variation in *MAPT* is not only associated with the dementia of PD but also differences in the neural circuitry underlying aspects of cognition in normal aging.

## 1 Introduction

Neurodegenerative diseases of the central nervous system represent a formidable challenge in our aging society. Parkinson's disease (PD) affects 1%–2% of the population over the age of 65 years, and although it is classically regarded as a movement disorder, associated cognitive impairment is a key determinant of quality of life and care requirements. Heterogenous mild cognitive deficits are present even at the time of diagnosis (Foltynie et al., 2004; Yarnall et al., 2014) and dementia affects 50%–80% of patients in the later disease stages (Aarsland et al., 2003; Williams-Gray et al., 2013). The executive cognitive deficits in PD are thought to be related to disturbances in frontostriatal dopaminergic systems (Williams-Gray et al., 2007), but there is also substantial evidence linking aspects of PD dementia to the progression of a “posterior cortical” syndrome of cognitive impairment (Williams-Gray et al., 2009) and the spread of Lewy body-type pathology into cortical and limbic structures (Aarsland et al., 2005).

Genetic association studies have identified the gene encoding the microtubule-associated protein tau, *MAPT*, as a key risk locus for several neurodegenerative disorders including PD (Caffrey and Wade-Martins, 2007). Tau is involved in the assembly and stabilization of microtubules but forms characteristic aggregates in several neurodegenerative diseases collectively known as the tauopathies. Although PD is not typically regarded as a tauopathy and is instead characterized by alpha-synuclein pathology, a link between *MAPT* and PD is now well established. Of the 2 common extended haplotypes (H1 and H2) that incorporate *MAPT*, there is overwhelming evidence that H1 is significantly overrepresented in PD patients relative to control subjects (Goris et al., 2007; Simón-Sánchez et al., 2009). In addition, it has emerged that these *MAPT* haplotypes are associated with the cognitive heterogeneity of PD. For example, our prospective study of a community-based PD cohort followed from diagnosis revealed that patients who are H1 homozygotes (H1/H1) had an accelerated rate of cognitive decline relative to H2 carriers (H1/H2, H2/H2) (Goris et al., 2007). These effects of *MAPT* interacted with age, such that the rate of cognitive decline was dependant on age in H1 homozygotes but not in H2 carriers (Goris et al., 2007). Over a 10 year follow-up period, *MAPT* H1 homozygosity continued to be one of the key predictors of dementia after adjustment for age (Evans et al., 2011; Williams-Gray et al., 2013). The same association has been reported in an independent cross-sectional study of 202 patients, in which there was a greater overrepresentation of the H1 haplotype among PD patients with dementia than those without (Setó-Salvia et al., 2011).

Despite compelling evidence that the common H1 versus H2 *MAPT* haplotypes are associated with differences in the presentation of PD, the relationship between haplotypic variation in *MAPT*, cognition, and underlying brain function remains relatively unexplored. To investigate the basis of the genotype-phenotype correlations, we used functional magnetic resonance imaging (fMRI) in a group of genotyped patients with PD and age-matched controls. Functional MRI has proven to be a sensitive indicator of presymptomatic dementia processes and has revealed altered patterns of brain function associated with genetic risk for Alzheimer's disease (AD) even in the absence of differences in cognitive function (Bondi et al., 2005; Bookheimer et al., 2000).

The cognitive task chosen for this study was a previously validated memory test involving picture encoding (Dove et al., 2006). We selected this task on the basis that (1) memory has been identified as one of the most commonly affected cognitive domains in PD (Aarsland et al., 2010; Yarnall et al., 2014); and (2) PD dementia has been hypothesized to develop at least in part from a syndrome of cognitive impairment characterized by deficits in posterior cortical function (Williams-Gray et al., 2009). We used event related fMRI to measure brain activation during different subcomponents of memory encoding, focusing our analysis on medial temporal lobe regions, particularly the hippocampus, which are known to be crucial for the registration of new information (Fernández et al., 1998; Milner et al., 1998). Our aims were to explore the relationship between the common *MAPT* haplotypes and memory function and to determine whether this relationship varied with the presence of PD and increasing age.

## 2 Methods

### 2.1 Participants

Data are presented from 77 right-handed participants who underwent MRI scanning at the Medical Research Council (MRC) Cognition and Brain Sciences Unit, Cambridge (Table 1). All participants were asked to abstain from caffeine for at least 3 hours and alcohol for 12 hours before the scan. They were offered a monetary reimbursement for their time at standard local MRC rates (£10/h) with a contribution toward travel costs. On the day of scanning, all participants completed the Addenbrooke's Cognitive Assessment Revised Version (ACE-R; Mioshi et al., 2006) incorporating the Mini-mental State Examination (Folstein et al., 1975); National Adult Reading Test (NART; Nelson, 1982) as an estimate of premorbid IQ; and the revised Beck Depression Inventory (BDI; Beck et al., 1961). Participants were required to have no significant subjective or objective cognitive deficit (Mini-mental State Examination score ≥26), no history of head trauma, and no major depression (BDI ≤18). The study was approved by the Cambridgeshire 2 Research Ethics Committee, UK (LREC number: 08/H0308/355) and the Addenbrooke's Research and Development Department. Written informed consent was obtained from all volunteers.

Patients were recruited via the PD research clinic at the John van Geest Centre for Brain Repair (BRC). All fulfilled the PD Society Brain Bank Criteria for idiopathic PD (Gibb and Lees, 1988) with mild-moderate disease (HYS ≤3) (Hoehn and Yahr, 1967) and remained on their usual medications during testing. Each patient's dopaminergic drug regime was converted to an equivalent L-dopa dose (Williams-Gray et al., 2007): Equivalent L-dopa dose = (L-dopa [× 1.2 if 10 mg selegiline OR × 1.1 if 5 mg selegiline]) + (pramipexole × 400) + (ropinirole × 40) + (cabergoline ×160) + (pergolide × 200) + (bromocriptine × 10) + (lisuride × 160) + (rasagiline × 100), all doses in mg. Patients' motor features were assessed on the day of scanning by a single assessor using Section 3 of the MDS-UPDRS (Goetz et al., 2008).

Control subjects were recruited from the volunteer panel at the MRC-CBU and via local advertisement and were screened for past or current neurologic problems.

### 2.2 Genotyping

Potential participants provided either a saliva sample via a postal Oragene kit (DNA Genotek Inc, Ontario) or a venous blood sample for genetic analysis before invitation for the scanning phase of the study and were selected on the basis of genotype. DNA was extracted from venous blood samples using standard salting out methods or from saliva samples according to the manufacturer's instructions. Genotyping for rs9468 (tagging *MAPT* H1 vs. H2 haplotype) was performed using a Taqman allelic discrimination assay and run on an HT7900 detection system (Applied Biosystems) (Goris et al., 2007).

### 2.3 Experimental paradigm

**Fig. 1:**
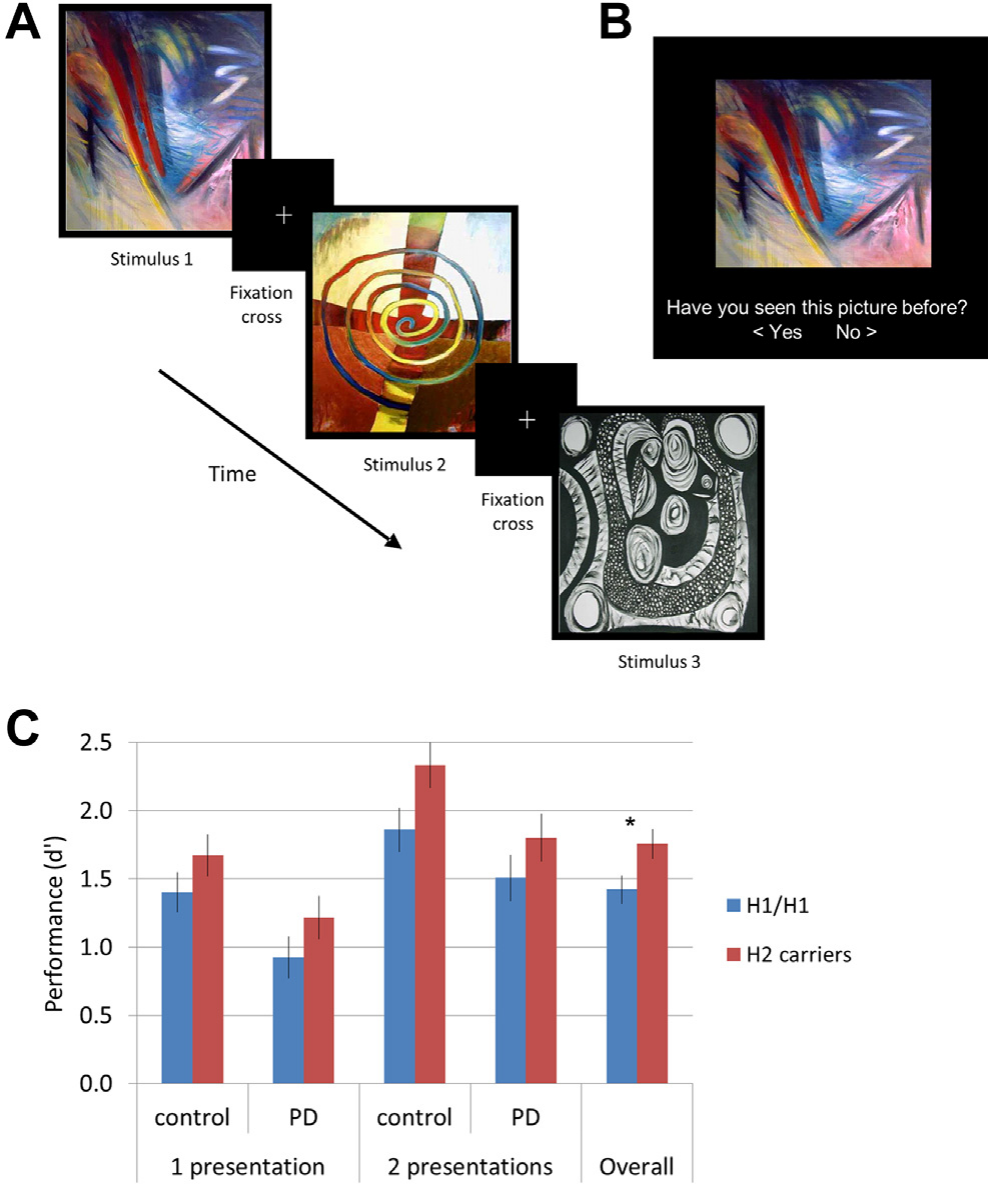
The abstract pictures memory encoding task. (A) A typical series of stimuli. (B) A typical presentation in the post-scanner test of recognition. Participants responded using the arrow keys on the keyboard. (C) Performance on the post-scanner test of recognition. d’ scores are shown (higher score represents greater discrimination during recognition memory). Group means are plotted. Error bars indicate ± 1 SEM. Asterisk denotes *p* < 0.05 based on main effects of repeated measures ANOVAs. There were additional significant effects of the number of presentations and disease status (PD patients vs. control subjects). Abbreviations: ANOVA, analysis of variance; PD, Parkinson's disease; SEM, standard error of the mean.

Participants viewed a series of abstract pictures in the scanner (Fig. 1A) and were asked to commit them to memory. Pictures were presented for 4 seconds in blocks of 6, with a fixation shown for 1 second between pictures and 20 seconds between blocks. Participants viewed a total of 36 different pictures in the scanner, 18 of which were then presented twice. Participants were not required to make any responses during the scan. Memory for the pictures was tested approximately 20 minutes after the presentation of the pictures in the scanner (Fig. 1B). During the memory test, a series of 72 pictures were shown, half of which had been displayed at least once in the scanner and the remainder of which were novel distractor pictures. Participants were asked to indicate whether they had seen each picture before.

### 2.4 Behavioral data analysis

For demographic and clinical characteristics, 1-way analyses of variance (ANOVAs) and χ^2^ tests were used to assess between-group differences in quantitative and categorical variables, respectively. Student *t* tests (2 tailed) were then used to compare the groups (PD vs. controls; H1 homozygotes vs. H2 carriers).

The behavioral outcome measure for the study was the number of pictures correctly classified as having been previously seen. As this task required participants to select one of 2 responses for each image, d’ values were calculated for each individual to take into account the relative proportions of true and false hits, therefore giving a measure of performance independent of response bias (Dove et al., 2006). Outliers (n = 2) were identified based on Tukey's Hinges percentile data and transformed using winsorization. Shapiro-Wilks tests confirmed that the scores within each group were normally distributed. The d’ values were entered into a repeated measures ANOVA with the number of previous presentations (1 vs. 2) as a within-subjects factor, and disease (patients vs. controls) and genetic group (H1 homozygotes vs. H2 carriers) as between-subject factors. Significant main effects and interactions were explored using least significant difference tests and Student *t* tests.

Data analyses were performed using the Statistical Package for Social Sciences (version 21.0: SPSS, Chicago IL), and the threshold for statistical significance was considered to be *p* ≤ 0.05. Where Mauchly test indicated that the assumptions of sphericity had been violated, Greenhouse-Geisser estimates were used.

### 2.5 Imaging acquisition

All pictures were acquired on a Siemens 3T Tim Trio Scanner (Siemens Medical Systems, Erlangen, Germany) with a 12-channel head coil. A T1-weighted structural image was acquired for each participant using a high resolution MPRAGE sequence (Repetition time = 2250 ms, Echo time = 2.99 ms, flip angle = 9°, field of view = 256 mm × 240 mm × 192 mm, voxel size = 1 mm isotropic). Structural scans of all participants were screened for gross abnormalities by a radiologist.

T2-weighted echo-planar pictures depicting blood-oxygen level-dependent (BOLD) signals were acquired using a standard Siemens EPI sequence for fMRI. The first 10 pictures (20 seconds) were discarded to avoid T1-equilibrium effects. Each image consisted of 32 slices of 3 mm thickness with a 25% interslice gap, with an in-plane resolution of 3 × 3 mm. The repetition time was 2 seconds. Slices were angled away from the orbits to avoid signal dropout because of magnetic susceptibility inhomogeneity. Stimuli were presented on a computer screen with a resolution of 1024 pixels which was visualized using a mirror positioned within the scanner at a viewing distance of 90 mm, such that 37 pixels subtended a visual angle of 1°.

### 2.6 MRI preprocessing

Imaging data were preprocessed using Statistical Parametric Mapping software (SPM8; Wellcome Department of Imaging Neuroscience, UCL, UK) implemented in MATLAB (MathWorks). SPM8 processing was implemented by the “aa” batch system (http://imaging.mrc-cbu.cam.ac.uk/imaging/AutomaticAnalysis). Structural MRI data were used for spatial normalization of the fMRI data and also for voxel-based morphometric analysis of brain structure. In the voxel-based morphometric analysis, gray matter volume was extracted for each participant from hippocampal, fusiform, and parahippocampal gyri regions of interest defined using the Automatic Anatomical Labeling atlas (AAL; Tzourio-Mazoyer et al., 2002), and these volumes were compared between-groups using linear models (see Supplementary Materials).

For each participant, fMRI volumes were motion corrected, slice time acquisition corrected, co-registered to the structural MPRAGE, segmented and normalized to the standard Montreal Neurological Institute echo-planar imaging template using the SPM8 unified segmentation, and smoothed with an 8 mm full width at half maximum Gaussian kernel. Six subject-specific movement predictor functions consisting of rotations and translations in the X, Y, and Z planes were extracted from this process, and the root mean squared of the “scan to scan” differences were calculated for each participant, representing the average displacement in space. Comparison of these values using repeated measures ANOVA with a within-subjects factor (parameter) and 2 between-subjects factors (PD patients vs. controls and *MAPT* H1 homozygotes vs. H2 carriers) confirmed no differences in motion associated with PD or *MAPT* haplotype.

### 2.7 Single-subject level fMRI analysis

Events during scanning were categorized into 3 types, namely: (1) presentation of pictures that were shown only once; (2) first presentation of pictures that were shown twice; and (3) second presentation of pictures that was shown twice. For each individual, every image was further categorized according to whether it was subsequently recognized in the post-scanner test (“remembered”) or not (“non-remembered”). The factorial design of event type (3) and subsequent memory (2) resulted in 6 conditions.

Fixed effects analyses were carried out at the single subject level. Whole brain activation maps were generated for each participant for the 6 conditions. Event onsets related to the time at which the stimulus appeared on the screen and durations related to the length of time for which the stimulus was shown. These onset and duration timings were convolved with a canonical haemodynamic response function to account for the lag between neural activity and the BOLD signal. Individual participant models included the 6 subject-specific movement predictor functions (see previously mentioned data). For each individual, 2 contrasts were made. The first contrast consisted of “remembered” minus “non-remembered” pictures and included only pictures that were shown once in the scanner to achieve one-to-one mapping between viewing the picture and subsequent recognition. The aim of this contrast was to identify regions that were specifically involved in successful memory encoding (Kim, 2011). The second contrast consisted of all events minus baseline and allowed us to look at more general differences associated with engagement in the task that would occur across both successful and unsuccessful trials.

### 2.8 Group level fMRI analysis

We performed a hierarchical series of analyses of the fMRI data based on our anatomically constrained hypotheses and the anticipated modest effect size of a single genetic variant on activation. First, we tested the hypothesis that differences associated with memory function would be seen in the hippocampus by means of a region of interest (ROI) analysis. Second, we performed a whole-brain voxel wise analysis with correction applied, to identify clusters of activation differences within the temporal lobes. Finally, we performed an exploratory whole-brain analysis to facilitate meta-analysis.

#### 2.8.1 Hippocampal ROI analysis

Mean activation data from hippocampal ROIs, defined using the AAL atlas, were extracted from each participant using the MarsBaR toolbox for each of the 2 contrasts (Brett et al., 2002). Data were then winsorised at 2.5 SDs and then subjected to a 2 × 2 × 2 ANOVA in which the between-subject factors were *MAPT* haplotype (H1 homozygotes vs. H2 carriers) and PD (patients vs. control subjects) and the within-subject factor was hemisphere (left vs. right).

#### 2.8.2 Temporal lobe clusters of activation and whole brain analyses

We then performed additional voxel-wise analyses, to detect activation differences outside the hippocampus as well as regional differences within the hippocampus that may have been obscured by averaging across the whole ROI. We used robust nonparametric cluster-based permutation analysis as implemented in the Cambridge Brain Activation software (CamBA v2.0.0; www-bmu.psychiatry.cam.ac.uk/software/; Bullmore et al., 1999; Suckling and Bullmore, 2004). Specifically, individual subjects' activation maps for each of the 2 contrasts were entered into voxel-wise ANOVAs. The first model included only PD patients with *MAPT* haplotype (H1 homozygotes vs. H2 carriers) as a between subjects factor. Age, sex, equivalent L-dopa, MDS-UPDRS motor score, and laterality of motor features (left vs. right) were then added as covariates. The second model included only controls, with *MAPT* haplotype (H1 homozygotes vs. H2 carriers) as a between subjects factor, and the third included PD patients and control subjects, with PD (patients vs. control subjects) and *MAPT* haplotype (H1 homozygotes vs. H2 carriers) as between subject factors. Regions activated during each of the 2 contrasts of interest were first identified by conducting a second-level group analysis across all 77 subjects. Linear contrasts were then defined to compare activation between groups.

We constructed an inclusive mask from all the temporal lobe anatomic regions defined in the AAL templates, to target our analysis on regions pertinent to memory. To stringently control for multiple comparisons, a cluster-based family wise error correction was applied in CamBA, such that the probability of one or more false positive clusters within the entire temporal lobe mask was *p* < 0.05 (for detailed description see Bullmore et al., 1999; Suckling and Bullmore, 2004). This cluster-based correction was applied to voxels that exceeded an uncorrected threshold of *p* < 0.05 in a whole-brain voxel wise analysis. The resultant statistical maps showing clusters that survived cluster correction (*p* < 0.05 FWE corrected) were rendered onto an MNI-space template.

Finally, in recognition of the potential contributions from regions outside of the temporal lobes, and with an aim to support future meta-analyses, we conducted an additional exploratory whole-brain analysis using an uncorrected voxel-wise threshold of *p* < 0.01. Full-factorial models were constructed as described previously, and the peak coordinates of activation differences associated with *MAPT* haplotype are reported in the Supplementary Materials. We note that our main inferences do not rely on these exploratory tests.

#### 2.8.3 Correlations with age

Correlations between age and activation were explored by extracting mean activation data from spherical ROIs with a 5 mm radius placed at the peak activation coordinates (left and right hippocampus) from our cluster-based analysis of contrast 1 (remembered minus non-remembered items). These ROIs were selected as, by the nature of the contrast, they are likely to represent key regions associated with successful task performance. Activation data were averaged across all voxels within the ROIs and Pearson correlations between activation and age performed in SPSS. To test for differences between the correlations in the groups, each correlation coefficient was converted into a z-score using Fisher r-to-z transformation. Then, making use of the sample size used to obtain each coefficient, these z-scores were compared using formula 2.8.5 from Cohen and Cohen (1983) via the online application available at http://quantpsy.org (Preacher, 2002).

## 3 Results

### 3.1 Association between MAPT haplotype and behavioral performance

Participants in all 4 groups (PD × *MAPT* haplotype) were matched in terms of overall cognitive performance on the ACE-R (Table 1); specifically, there were no between-group differences on the memory subsection of this assessment (*F* = 0.39, *p* = 0.760). NART and BDI scores differed between patients and control subjects, and these scores were therefore added as covariates in behavioral analyses. There were, however, no significant differences between H1 homozygotes and H2 carriers regardless of disease status.

Across all participants, pictures were categorized as previously seen or unseen more accurately than would be predicted by chance (1 sample *t* test: d’ scores vs. “0,” *p* < 0.001) (Fig. 1C). As expected, there was a significant effect of the number of presentations (*F*[1,73] = 106.65, *p* < 0.001), with enhanced memory for pictures shown twice. Critically, there was a significant main effect of *MAPT* haplotype (*F*[1,73] = 4.95, *p* = 0.029) with H2 carriers outperforming H1 homozygotes. Overall, controls made more correct responses than PD patients (*F*[1,73] = 9.23, *p* = 0.003); however, there was no interaction between PD and *MAPT* haplotype on performance (*F*[1,73] = 0.66, *p* = 0.798). Inclusion of sex and age as covariates revealed an additional effect of sex (*F*[1,71] = 5.38, *p* = 0.023) which corresponded to better performance in females than males, but no main effect of age (*F*[1,71] = 0.17, *p* = 0.684). The significant effects of *MAPT* haplotype and PD remained after addition of NART and BDI (adjusted to group means) as covariates. Separate ANOVAs in the patients and control subjects showed that the effects of *MAPT* haplotype were below the threshold for significance in the 2 groups (controls *F*[1,38] = 3.23, *p* = 0.080; PD patients *F*[1,35] = 1.85, *p* = 0.183).

### 3.2 Association between MAPT haplotype and BOLD response during successful encoding

**Fig. 2:**
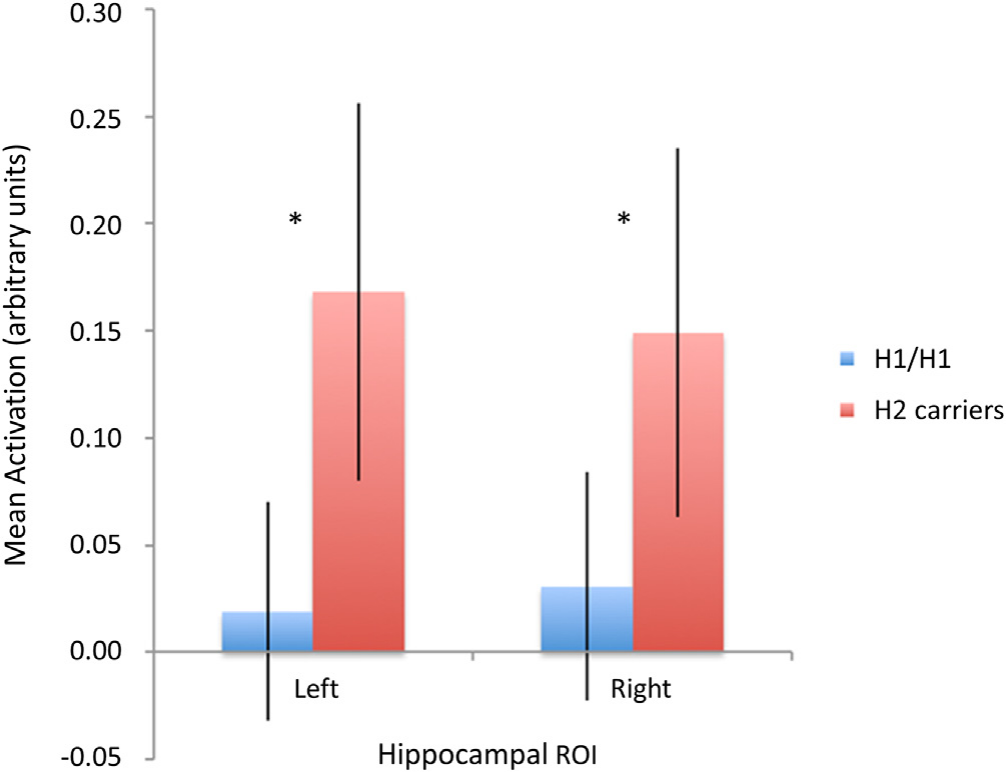
Activation in the hippocampal regions of interest (ROI) during successful encoding (contrast 1: successful minus unsuccessful encoding) in all participants stratified by *MAPT* haplotype. ROIs were defined anatomically using AAL templates, and activation is expressed in arbitrary units using the mean parameter estimates from the ROI. *p < 0.05 (2-tailed *t* tests). Error bars indicate ± 1 SEM. Abbreviations: AAL, anatomical labeling atlas; PD, Parkinson's disease; SEM, standard error of the mean.

In our first contrast, we looked at activation during non-remembered events subtracted from remembered events to establish activation patterns associated with successful encoding. Mean activation data were first extracted from the bilateral anatomically predefined hippocampal ROIs. An averaged *t* contrast suggested an overall involvement of this region in successful encoding; however, this did not reach significance (left hippocampus: *t* = 1.39, *p* = 0.084; right hippocampus: *t* = 1.30, *p* = 0.099). A 2 × 2 × 2 ANOVA (PD × *MAPT* × hemisphere) did however reveal a significant main effect of *MAPT* haplotype on activation in the hippocampus bilaterally in both patients and controls (*F* = 4.00, *p* < 0.05). Two-tailed Student t tests confirmed that this corresponded to significantly lower activation in H1 homozygotes than in H2 carriers in the right (*t* = 1.84, *p* = 0.035) and left (*t* = 2.22, *p* = 0.015) hippocampal ROIs (Fig. 2). There was no main effect of PD no significant additive effects of PD and *MAPT* haplotype nor any interactions (all *p* > 0.4).

**Fig. 3:**
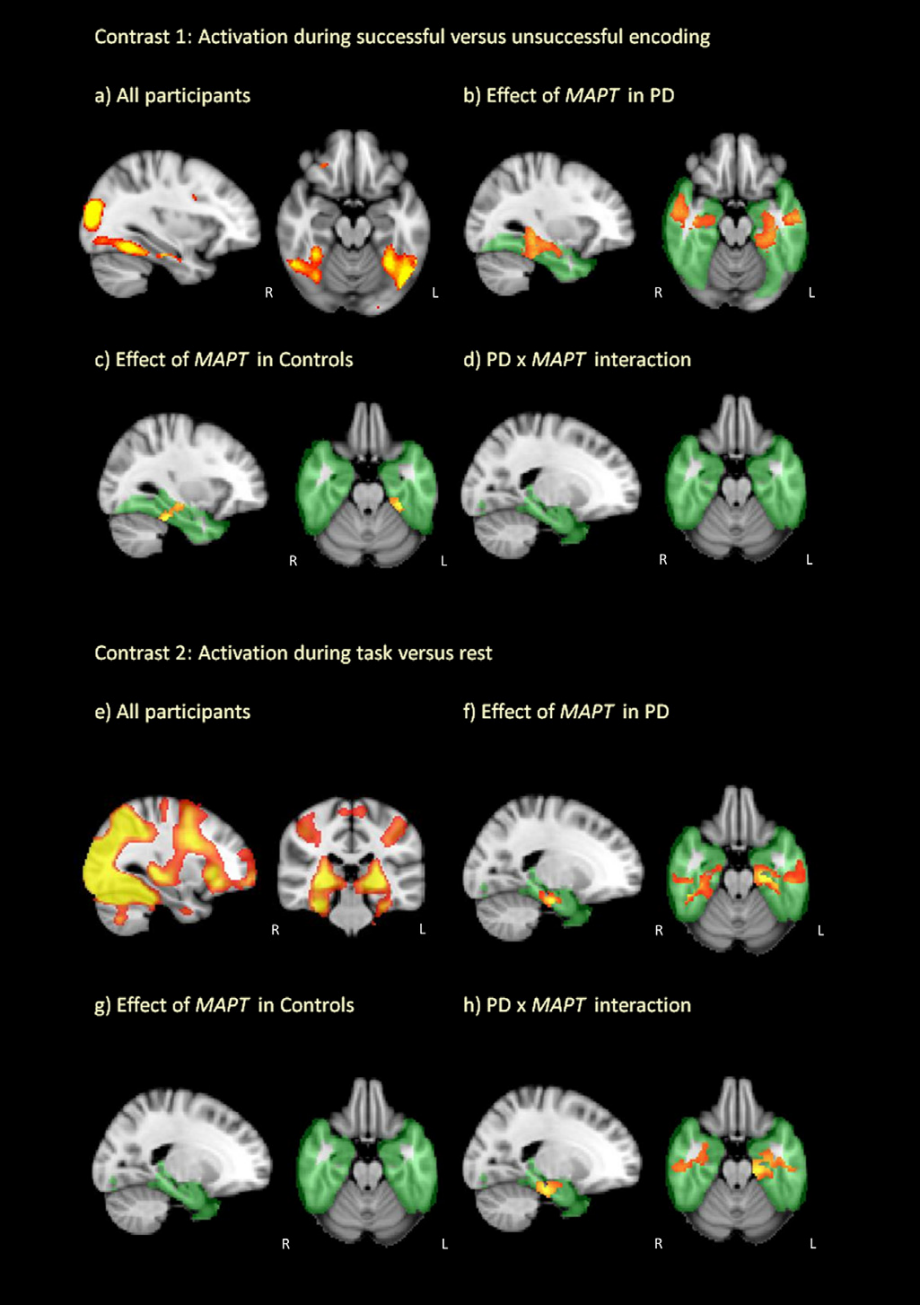
BOLD activations associated with performance of the memory encoding task. (A) Contrast 1: Activation during successful encoding (i.e. remembered minus non-remembered pictures) across all participants. (B) Effect of *MAPT* haplotype on Contrast 1 activation in patients with PD. (C) Effect of *MAPT* haplotype on Contrast 1 activation in control subjects. (D) Interaction between *MAPT* haplotype and PD (PD patients vs. control subjects) on Contrast 1 activation. (E) Contrast 2: Activation during overall task performance minus rest across all participants. (F) Effect of *MAPT* haplotype on Contrast 2 activation in patients with PD. (G) Effect of *MAPT* haplotype on Contrast 2 activation in control subjects. (H) Interaction between *MAPT* haplotype and PD (PD patients vs. control subjects) on Contrast 2 activation. All effects of *MAPT* correspond with increased activation in H2 carriers compared with H1 homozygotes (H1/H1). Activation that survived permutation-based cluster correction (*p* < 0.05 FWE corrected) within a predefined inclusive mask of the temporal lobe region of interest (green) is displayed. Abbreviations: BOLD, blood-oxygen level-dependent; PD, Parkinson's disease.

Voxel-wise analysis of this contrast showed that successful memory encoding was associated with extensive activation in the medial temporal lobes bilaterally (Fig. 3A). Among the PD patients, H1 homozygotes had less activation than H2 carriers across the hippocampus, inferior temporal lobes, fusiform, and parahippocampal gyri bilaterally at the cluster-corrected threshold (*p* < 0.05 FWE corrected; Fig. 3B; Table 2). These effects remained, and no other significant effects or interactions were found, after the addition of covariates. Analyzing control data revealed similar differences associated with *MAPT* haplotype with H1 homozygotes having less activation than H2 carriers especially within a left temporal lobe cluster incorporating the hippocampus (Fig. 3C; Table 2). Including patients and control subjects in a 2 × 2 full factorial design (PD × *MAPT*) showed no main effect of PD nor any other significant effects or interactions (Fig. 3D). Examination of data extracted from peak activation clusters revealed no correlations between activation and age in any of the patient or control *MAPT* haplotype subgroups.

### 3.3 Association between *MAPT* haplotype and BOLD response during overall performance

In our second contrast, all events were averaged to establish activation patterns associated with overall task performance relative to rest. Mean activation data were first extracted from the bilateral anatomically defined hippocampal ROIs. An averaged *t* contrast showed that there was a strong hippocampal activation in this task (left hippocampus: *t* = 3.99, *p* < 0.001; right hippocampus: *t* = 4.12, *p* < 0.001). A 2 × 2 × 2 ANOVA (PD × *MAPT* × hemisphere) did not reveal any significant effects of *MAPT* haplotype or PD, nor any interactions.

Voxel-wise analysis of this contrast revealed that general engagement in the task was associated with significant activation across a network of regions including the visual processing areas, frontoparietal cortices, and the medial temporal lobes (Fig. 3E). In patients with PD, there was a significant effect of *MAPT* haplotype with less activation in H1 homozygotes than H2 carriers bilaterally in the hippocampus, inferior temporal lobe, fusiform, and parahippocampal gyri at the cluster-corrected threshold (*p* < 0.05 FWE corrected; Fig. 3F; Table 2). Addition of covariates revealed no other significant effects of L-dopa, motor scores, or disease lateralization. In contrast, the control subjects did not exhibit a significant effect of *MAPT* haplotype within the medial temporal lobes (Fig. 3G). Including patients and control subjects in a 2 × 2 full factorial model revealed no main effect of PD or *MAPT* haplotype; however, there was a significant interaction between *MAPT* haplotype and PD, supporting the view that there were differential effects of *MAPT* haplotype in PD patients and control subjects (Fig. 3H; Table 2).

**Fig. 4:**
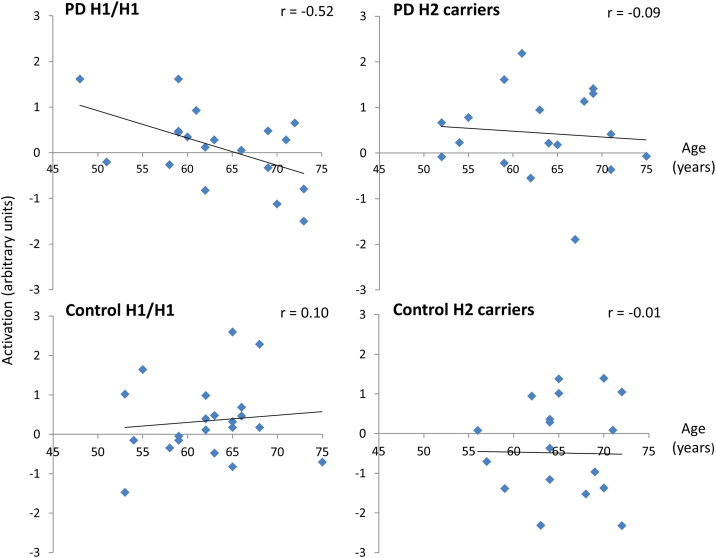
Correlations between age and hippocampal activation during overall task performance. Mean activation data for contrast 2 (“all events minus baseline”) was extracted from peak activation coordinates defined in contrast 1 (“successful encoding”). Values are plotted after winsorizing one outlier (>2.5 SD from mean) in the group of H1 homozygotes (H1/H1) with Parkinson's disease (PD). A significant negative correlation (*p* < 0.05) between age and activation is seen in H1 homozygotes with PD. Abbreviation: SD, standard deviation.

To explore the effects of age on overall task-related activity, data from the peak activation clusters in the hippocampus (defined in contrast 1) were extracted. Only within the group of PD H1 homozygotes was there a significant negative correlation between activation and age (*r*[17] = −0.61, *p* < 0.01), with the correlations in the other 3 groups being close to zero (all |r| ≤ 0.1; Fig. 4). One H1 homozygote with PD had a value which was more than 2.5 SDs from the mean; however, the correlation between age and activation remained significant in this group after replacing this outlier with the nearest value in the data set (*r*[17] = −0.52, *p* < 0.01). Fisher tests showed that the negative correlation in the PD H1 homozygotes differed significantly from the correlation collapsed across all other groups (z = −2.25, *p* = 0.024 two tailed). Comparing the groups individually, the correlation in the PD H1 homozygotes differed significantly from the correlations in the control groups (control H1/H1: z = −2.36, *p* = 0.018; control H2 carriers: z = −0.198, *p* = 0.048) and with a trend difference from the correlation in the PD H2 carriers (z = −1.72, *p* = 0.085).

## 4 Discussion

The aim of this study was to define differences associated with common haplotypic variation in *MAPT* during memory formation in patients with PD and healthy age-matched control subjects. Brain activation was measured using fMRI as participants intentionally memorized abstract pictures and their ability to remember these pictures was subsequently assessed in a test of recognition. To our knowledge, this is the first study to investigate the neurocognitive correlates of haplotypic variation in *MAPT* in patients with PD and control subjects using fMRI, and we anticipate that our findings will contribute to our understanding of the relationship between this common genetic variant, cognitive dysfunction, and dementia in PD.

Behaviorally, we found that across a group of 77 nondemented patients with PD and age-matched controls, *MAPT* H1 homozygotes had a poorer ability to recognize pictures seen in the scanner than their H2 carrier counterparts. The effects of *MAPT* haplotype did not reach significance in the PD and control subgroups when analyzed independently; however, there were no interactions between *MAPT* haplotype and PD, suggesting that this effect was consistent regardless of disease status. These differences occurred in the absence of differences in global cognitive function between genetic groups as measured using the ACE-R. Our finding of an association between *MAPT* haplotypes and specific memory deficits is in agreement with the results of a cohort study of 212 patients with PD and mean disease duration of over 6 years, in which H1 homozygosity was consistently associated with selective impairments on the memory subscale of the Mattis Dementia Rating Scale (Morley et al., 2012). The direction of effects also links in with our previous observation of poorer cognitive outcome in H1 homozygotes with PD (Evans et al., 2011; Goris et al., 2007) and the well-established associations between H1 haplotype and risk for neurodegenerative diseases characterized by dementia, including progressive supranuclear palsy and corticobasal degeneration (Caffrey and Wade-Martins, 2007). Despite a lack of association between *MAPT* haplotype and the risk of developing AD, H1 homozygosity has been linked to an accelerated rate of conversion from mild cognitive impairment to AD dementia (Samaranch et al., 2010). Although it therefore appears that haplotypic variation in *MAPT* has wide-reaching associations with cognitive function, its relationship with cognitive progression in healthy aging is relatively unknown.

Our primary fMRI analysis revealed that activity in the medial temporal lobes during encoding varied, depending on whether or not an item was subsequently remembered. The association between increased medial temporal lobe activation during encoding and success at recall is well described (Brewer et al., 1998; Ranganath and D'Esposito, 2001), and the regions identified in our study are consistent with those identified in a meta-analysis of fMRI studies using similar subsequent memory approaches: inferior frontal cortex, bilateral fusiform cortex, bilateral hippocampal formation, bilateral premotor cortex, and bilateral posterior parietal cortex (Kim, 2011).

Here, we show that this relationship between activation during encoding and subsequent ability to recognize pictures varies as a function of *MAPT* haplotype, irrespective of whether or not participants had PD. More specifically, H1 homozygotes exhibited less recruitment of the medial temporal lobe than H2 carriers during encoding that was followed by successful recognition. On this basis, we propose that there is an uncoupling of the normal relationship between medial temporal lobe regulation and memory performance in this genetic group.

To explore this further, our second contrast looked at activation during the overall engagement in the task, that is, encoding of images irrespective of whether they were later recognized, in line with previous studies (Bookheimer et al., 2000). Here, we found that the differences associated with *MAPT* haplotype were not uniform across PD patients and control subjects. It was apparent that in the PD group, but not in the control subjects, H1 homozygotes had significantly less medial temporal lobe activation than H2 carriers. In studies of AD, hypoactivation and hyperactivation of the medial temporal lobe has been reported during memory tasks in individuals who are at risk of developing dementia. It has generally been suggested that increased hippocampal activation is a compensatory response that allows for the maintenance of relatively normal memory function in the face of developing pathologic change (Bondi et al., 2005; Bookheimer et al., 2000) and by inference, reduced activation may reflect superior neuronal efficiency. However, in situations such as that reported here, where the hypoactivation is coupled with impaired memory performance, an alternative interpretation is that the reduced medial temporal lobe activation may relate to the detrimental effects of neurodegenerative change on brain function (Machulda et al., 2003; Sperling et al., 2003).

We therefore postulate that the additional differences associated with *MAPT* haplotype in our PD group could reflect the influence of this genetic variant on a pathologic process leading to dementia. Medial temporal lobe abnormalities have been described in PD (Weintraub et al., 2012), and it is possible that *MAPT* haplotype alters the accumulation of pathology in these regions. Of particular relevance, therefore, was the significant negative correlation between age and task-related activation that we observed exclusively among the H1 homozygotes with PD. These age-dependent effects in H1 homozygotes coincide with our previous clinical findings of an interaction between age and *MAPT* haplotype on cognitive decline in PD, in which cognitive decline was age dependent in H1 homozygotes but more static in H2 carriers (Goris et al., 2007).

Our finding of impaired behavioral performance in PD patients relative to control subjects is consistent with previous reports of memory deficits in PD, even in patients without dementia (Aarsland et al., 2010; Yarnall et al., 2014). Interestingly, however, despite these differences in behavioral performance, we found no main differences associated with PD on activation in the temporal lobes during successful encoding. One possibility is that the differences in behavioral performance between PD patients and control subjects could be explained by a mechanism that is distinct from memory encoding per se. Of note, our behavioral outcomes and the designation of events during fMRI as being “remembered” or “non-remembered” were based on a post-scan assessment. It is possible that other factors contributed to performance in this assessment, and these may not have manifested as differences in activation which was recorded during picture presentation in the scanner. It has, for example, been proposed that some of the memory deficits in PD may be secondary to executive dysfunction, attentional impairments, and disordered internally cued search strategies related to frontostriatal dopamine disturbances. This has been supported by the observation that, in contrast to AD, impaired recall in PD may improve with cueing, suggesting that memory impairment relates to difficulties in the retrieval rather than the storage of information (Helkala et al., 1988; Pillon et al., 1993).

In response to the question of whether structural differences are contributing to the activation differences reported, we performed a voxel-based morphometry to compare gray matter volumes between groups in the regions in which activation differences were found (see Supplementary Material for details). There were differences associated with *MAPT* haplotype in the right hippocampus, and a trend in left hippocampus, but not the other regions analyzed. However, whereas H1 haplotype was associated with reduced activation, it was associated with increased gray matter volume, suggesting that the activation differences we observed with fMRI are not because of partial volume reductions in H1 haplotype carriers (i.e., hippocampal dysfunction but not simply hippocampal atrophy). The mechanism of the relationship seen between *MAPT* haplotype and brain function in this study remains to be determined. At a cellular level, there is accumulating evidence that *MAPT* haplotype influences tau transcription, with several studies reporting that H1 haplotype is associated with increased expression of total tau or of 4-repeat tau compared with the H2 haplotype (Kwok et al., 2004; Myers et al., 2007; Rademakers et al., 2005; Williams-Gray et al., 2009). The relative transcription of tau isoforms with 3 or 4 microtubule-binding domains (3- or 4-repeat tau) has been shown to be altered in PD (Tobin et al., 2008), and our own study demonstrated that the H1 haplotype was associated with a 20% increase in 4-repeat tau transcription in PD brains (Williams-Gray et al., 2009). Postmortem studies have supported the concept that *MAPT* H1 haplotype drives Lewy body pathology without any significant contribution to Alzheimer's type changes. In one study of 22 cases of dementia with Lewy bodies, total Lewy body counts and alpha-synuclein deposits were significantly higher among the H1 homozygotes than in the H2 carriers (Colom-Cadena et al., 2013). A postmortem series of 762 cases of AD, Lewy body diseases and vascular pathology linked H1 haplotype to reduced Alzheimer's type pathology and increased Lewy body counts (Wider et al., 2012), and it is conceivable that the effect of H1 on Lewy body accumulation may underlie the observation of accelerated cognitive decline in clinically defined AD cases in whom mixed pathology is often seen (Samaranch et al., 2010).

However, given our observation that *MAPT* haplotypes are also associated with differences in brain function in control subjects, it is possible that these genetic variants exert effects on brain function in aging more generally. These effects may either be related to emergent pathology—for example, the aforementioned accumulation of Lewy bodies—or could reflect a developmental influence, comparable with recent suggestions of the pleiotrophic effects of *APOE* polymorphisms in which the relative beneficial and detrimental effects of a genetic variant vary over an individual's lifetime (Dean et al., 2014). Further behavioral and postmortem studies in healthy control subjects and in different disease groups, at different ages, are needed to clarify whether these effects occur earlier in development or present only during aging.

Several limitations of this study should be considered. We looked at 2 genetic groups to specifically follow up previous studies which have reported differential effects in H1 homozygotes compared with H2 carriers (Goris et al., 2007; Williams-Gray et al., 2013); however, H2 homozygosity may be associated with a different pattern of effects which could be further explored. The patients were tested on L-dopa medication, however, critically, doses were matched between the genetic subgroups and included as covariates in the analysis, and should therefore not have impacted on our primary aim which was to evaluate the effects of *MAPT* haplotypes. Because of our a priori hypothesis, our analyses were primarily focused on the effects of *MAPT* haplotypes on temporal lobe regions. However, although there is evidence that PD dementia is linked to posterior cortical dysfunction, there are likely to be contributions from dysfunction across distributed brain regions which warrant future study.

In conclusion, these findings endorse the fact that common haplotypic variation in *MAPT* has important and clinically relevant associations with cognitive function in PD and also implicate this genetic variant in the function of the normal aging brain. We anticipate that these findings will have implications for our understanding of cognitive dysfunction in PD and also more broadly, with potential applications in risk stratification, prognostication, and the development of appropriately targeted-treatment strategies.

## Disclosure statement

The authors declare no actual or potential conflicts of interest. All participants gave written informed consent, and the study was approved by the local Regional Ethics Committee and Research and Development Department.

## Figures and Tables

**Table 1:** Clinical and demographic characteristics of the participants

Variable	Controls		PD patients		*p*-value of group differences		
	H1/H1	H2 carriers	H1/H1	H2 carriers	All groups	*MAPT*	PD vs. control
N	21	19	19	18	—	—	—
Sex (male: female)	13:8	11:8	11:8	12:6	0.94	0.85	0.85
Age (y)	62.6 ± 5.6	65.7 ± 4.8	64.0 ± 7.2	63.6 ± 7.0	0.45	0.31	0.81
ACE-R (%)	92.6 ± 2.4	95.8 ± 3.0	95.4 ± 3.8	94.9 ± 3.5	0.14	0.98	0.07
ACE-R memory (/26)	24.5 ± 1.9	24.0 ± 2.4	24.2 ± 1.9	23.7 ± 2.9	0.76	0.37	0.55
BDI	4.2 ± 4.3	5.5 ± 5.0	6.2 ± 3.0	8.7 ± 5.3	0.03	0.09	0.02
NART	122.4 ± 4.7	121.9 ± 6.4	117.9 ± 8.0	117.2 ± 8.3	0.05	0.69	0.01
Years of education	15.3 ± 3.5	15.1 ± 3.0	13.7 ± 2.9	13.9 ± 3.4	0.38	0.96	0.08
H&Y	NA	NA	1.8 ± 0.4	1.7 ± 0.5	0.65	0.65	NA
MDS-UPDRS 3	NA	NA	28.2 ± 11.0	28.0 ± 12.4	0.97	0.97	NA
Years since diagnosis	NA	NA	6.7 ± 2.4	6.8 ± 4.0	0.98	0.98	NA
Actual L-dopa (mg)	NA	NA	329.0 ± 231.7	361.9 ± 208.2	0.65	0.65	NA
Equivalent L-dopa (mg)	NA	NA	1202.6 ± 685.2	939.7 ± 526.4	0.20	0.20	NA

Values are expressed as group means ± standard deviations for continuous variables. *p* values are derived from 1-way ANOVAs comparing all 4 groups (PD H1/H1, PD H2 carriers, control H1/H1, control H2 carriers); 2-tailed *t* tests comparing groups by *MAPT* haplotype and disease presence; and χ^2^ tests for categorical variables. Results are presented without correction for multiple comparisons. Key: ACE-R, Addenbrooke's Cognitive Examination (revised version); ANOVA, analysis of variance; BDI, Beck Depression Inventory; H&Y, Hoehn and Yahr; MDS-UPDRS 3, Movement Disorders Society Unified Parkinson's Disease Rating Scale Section 3 (motor features); NA, measure not applicable; NART, National Adult Reading Test; PD, Parkinson's disease.

**Table 2:** Activation differences associated with *MAPT* haplotype during memory task performance

Contrast	Contrast activation areas	Cluster size (voxels)	Peak value coordinates		
			x	y	z
Contrast 1: successful encoding (remembered minus non-remembered)					
All participants; H2 carriers > H1/H1					
Cluster 1	L hippocampus, L parahippocampal gyrus, L amygdala, L fusiform gyrus, L inferior temporal gyrus	927	−36	−34	−8
Cluster 2	L Heschl's gyrus, L superior temporal gyrus, L middle temporal gyrus, L inferior temporal gyrus	1687	−48	−48	−4
Cluster 3	R hippocampus, R parahippocampal gyrus, R fusiform, R Heschl's gyrus, R superior temporal gyrus, R superior temporal pole, R middle temporal gyrus, R middle temporal pole, R inferior temporal gyrus	2831	42	−18	0
Patients with PD; H2 carriers > H1/H1					
Cluster 1	L hippocampus, L parahippocampal gyrus, L amygdala, L fusiform gyrus, L inferior temporal gyrus	619	−36	−32	−10
Cluster 2	R hippocampus, R parahippocampal gyrus, R amygdala, R fusiform gyrus, R inferior temporal gyrus	484	44	−16	−24
Controls; H2 carriers > H1/H1					
Cluster 1	L hippocampus, L parahippocampal gyrus, L fusiform gyrus	230	−30	−34	−26
Contrast 2: overall task performance (all events minus baseline)					
PD × MAPT interaction					
Cluster 1	L hippocampus, L parahippocampal gyrus, L amygdala, L fusiform gyrus, L inferior temporal gyrus	811	−20	−24	−20
Cluster 2	R hippocampus, R parahippocampal gyrus, R amygdala, R fusiform gyrus, R inferior temporal gyrus	259	58	−26	−16
Patients with PD; H2 carriers > H1/H1					
Cluster 1	L hippocampus, L parahippocampal gyrus, L fusiform gyrus, L superior temporal gyrus, L middle temporal gyrus, L inferior temporal gyrus	2102	−24	−20	−22
Cluster 2	R hippocampus, R parahippocampal gyrus, R fusiform gyrus, R superior temporal gyrus, R superior temporal pole, R middle temporal gyrus, R inferior temporal gyrus	2316	50	−38	−20
Controls; H2 carriers > H1/H1					
Cluster 1	R superior temporal gyrus, R middle temporal gyrus	582	40	−54	10

Details are provided for clusters of activation surviving cluster-based correction for multiple comparisons within an inclusive mask of the temporal lobes (*p* < 0.05 FWE corrected). There were no significant interactive effects of *MAPT* haplotype (H1 homozygotes vs. H2 carriers) and PD (patients vs. controls) for contrast 1 and no significant effect of *MAPT* haplotype (H2 carriers > H1/H1) across all participants for contrast 2. Coordinates listed are in Montreal Neurological Institute (MNI) standard space. Anatomic labels are derived from the AAL atlas. Key: FWE, Family-wise error; L, left; PD, Parkinson's disease; R, right.
