# Supplementary material for: Association between MAPT haplotype and memory function in patients with Parkinson's disease and healthy aging individuals
Source: Neurobiol Aging. 2015 Mar;36(3):1519–28. doi: 10.1016/j.neurobiolaging.2014.12.006 (PMC4353560; doi:10.1016/j.neurobiolaging.2014.12.006)
Supplement: Supplementary Material [file mmc1.docx]

**Association between *MAPT* haplotype and memory function in Parkinson’s disease and healthy aging**

Sophie E Winder-Rhodes, Adam Hampshire, James B Rowe, Jonathan E Peelle, Trevor W Robbins, Adrian M Owen, Roger A Barker

# Supplementary methods and results

#### Voxel Based Morphometric Structural Analysis

Data from the structural images were pre-processed and analysed in SPM8 (Wellcome Trust Centre for Neuroimaging, London, UK, <http://www.fil.ion.ucl.au.uk/spm>) using aa version 3.01 scripts (<http://www.cambridgeneuroimaging.com/aawiki/>). Prior to segmentation, bias-corrected structural images were created to reduce the influence of intensity inhomogeneity on segmentation; producing a separate bias-corrected image effectively results in a two-pass bias correction, as bias correction is also included in the segmentation process. Each individual’s structural image was first co-registered to an MNI-space average template distributed with SPM8 using normalized mutual information. This ensured reasonable starting estimates for the unified segmentation routine, and was done as an alternative to manually repositioning each scan. Structural images were then segmented into tissue classes using unified segmentation (Ashburner and Friston, 2005) as implemented in the “new segment” option of SPM8. The volume of the resulting grey matter, white matter, and CSF tissue classes was determined from the (unsmoothed, unregistered) segmented images by integrating over all voxels and multiplying by voxel size, and the volumes of these three classes were summed to provide an estimate of total intracranial volume (TIV). The tissue class images created during segmentation were then used to generate a custom template using DARTEL, a diffeomorphic registration method (Ashburner, 2007; Ashburner and Friston, 2009). During spatial normalization, images were modulated to preserve the total grey matter volume, and smoothed using an 8 mm FWHM isotropic Gaussian kernel.

Smoothed, modulated GM volume maps were compared between groups on a voxel-wise basis using the General Linear Model based on Gaussian field theory. Analyses of variance (ANCOVA) were set up; since a correction for volume change induced by spatial normalization had previously been incorporated into the modulation, TIV was included as a covariate of no interest in the analysis to remove any variance due to head size (Barnes *et al.*, 2010). In addition, age and sex were included as covariates. Total gray matter volume was not included as a covariate, and thus results reflect differences in actual gray matter volume (Peelle *et al.*, 2012). Data from pre-defined anatomical regions of interest (hippocampus, parahippocampal gyrus and fusiform gyrus defined using the Automatic Anatomical Labeling atlas (AAL; Tzourio-Mazoyer *et al.*, 2002) were extracted using MarsBaR (Brett *et al.*, 2002) and linear contrasts (F-tests) were defined to test for differences in GM volumes between the groups. A Bonferroni correction was applied to p values to take into account the number of regions in the analysis.

There was a significant effect of *MAPT* haplotype in the right hippocampus (F = 9.08; uncorrected p = 0.004, corrected p = 0.021) across the whole group, and t-tests confirmed that there was significantly higher right hippocampal GM volume in H1 homozygotes than in H2 carriers. There was a subthreshold trend in the same direction in the left hippocampus (F = 3.31, uncorrected p = 0.073, corrected p = 0.366). There were no significant differences associated with *MAPT* haplotype in the fusiform or parahippocampal gyri, neither were there any significant effects of PD or interactions between PD and *MAPT* haplotype in any region.

**Supplementary Tables: Whole brain analyses**

| **Contrast 1: Successful encoding (remembered minus non-remembered) ^a^** | | | | | | |
| --- | --- | --- | --- | --- | --- | --- |
|  | **Cluster Extent (voxels)** | **Peak t** | **Coordinates {mm}** | | | **Location** |
|  |  |  | **x** | **y** | **z** |  |
| All participants H2 carriers  > H1/H1 | 834 | 3.5 | -2 | 8 | 4 | L anterior cingulate, L caudate  R anterior cingulate, R caudate |
|  | 835 | 3.36 | 40 | -20 | 2 | R insula |
|  | 53 | 3.35 | -58 | 20 | 14 | L inferior frontal gyrus |
|  | 305 | 3.29 | 14 | 6 | 28 | R caudate, R middle cingulate |
|  | 132 | 3.16 | -36 | -16 | 0 | L superior temporal gyrus |
|  | 124 | 2.84 | -32 | 10 | 16 | L insula |
|  | 24 | 2.79 | -48 | -48 | -4 | L middle temporal gyrus |
|  | 15 | 2.72 | 44 | -14 | -22 | R fusiform gyrus, R inferior temporal gyrus, R hippocampus |
|  | 15 | 2.7 | 68 | -30 | 20 | R superior temporal gyrus |
|  | 33 | 2.69 | 26 | -28 | 16 | R thalamus, R insula |
|  | 28 | 2.66 | 22 | 24 | -12 | R orbitofrontal cortex |
|  | 33 | 2.65 | 54 | 2 | 18 | R rolandic operculum |
|  | 29 | 2.59 | -26 | -18 | -8 | L hippocampus |
|  | 18 | 2.58 | 22 | -44 | 42 | R precuneus |
| PD H2 carriers > H1/H1 | 92 | 3.39 | 44 | -14 | -22 | R hippocampus, R inferior temporal gyrus, R fusiform gyrus |
|  | 619 | 3.3 | 16 | 8 | 26 | R caudate |
|  | 336 | 3.28 | -4 | 24 | -2 | L anterior cingulate |
|  | 81 | 3.17 | 20 | 28 | -12 | R orbitofrontal cortex |
|  | 275 | 3.1 | -32 | 12 | 18 | L insula |
|  | 267 | 3.07 | 34 | -22 | 44 | R postcentral gyrus |
|  | 277 | 3.05 | 36 | -20 | 0 | R superior temporal gyrus |
|  | 43 | 3.02 | -56 | -8 | -26 | L middle temporal gyrus |
|  | 60 | 2.92 | -18 | -10 | 40 | L middle cingulate |
|  | 23 | 2.89 | -24 | 24 | -18 | L orbitofrontal frontal cortex |
|  | 47 | 2.68 | 18 | -42 | 44 | R precuneus |
|  | 29 | 2.67 | 36 | 26 | 20 | R middle frontal gyrus |
|  | 55 | 2.58 | -42 | -32 | -2 | L hippocampus |
|  | 17 | 2.55 | -38 | -16 | -18 | L hippocampus, L fusiform |
| Controls H2 carriers  > H1/H1 | 525 | 3.51 | -2 | 8 | 6 | L caudate, R caudate |
|  | 37 | 2.68 | 42 | -16 | 18 | R rolandic operculum |
|  | 16 | 2.57 | 26 | -30 | 18 | R insula |
|  | 15 | 2.52 | 44 | -4 | 0 | R superior temporal gyrus |

| **Contrast 2: Overall task performance (all events minus baseline) ^b^** | | | | | | |
| --- | --- | --- | --- | --- | --- | --- |
| **Contrast** | **Cluster**  **extent (voxels)** | **Peak t** | **Coordinates {mm}** | | | **Location** |
|  |  |  | **x** | **y** | **z** |  |
| All participants H2 carriers > H1/H1 | 144 | 3.24 | 22 | -94 | 12 | R cuneus |
|  | 62 | 2.87 | 26 | -56 | 40 | R angular gyrus |
|  | 55 | 2.75 | -34 | -84 | 6 | L middle occipital lobe |
|  | 47 | 2.69 | -14 | -92 | 10 | L superior occipital lobe |
| PD H2 carriers  > H1/H1 | 153 | 2.77 | -52 | -28 | -12 | L middle temporal gyrus |
|  | 27 | 2.59 | 56 | -20 | -12 | R middle temporal gurus |
| Controls H2 carriers > H1/H1 | 218 | 3.86 | 20 | -96 | 10 | R superior occipital lobe, R cuneus |
|  | 99 | 3.01 | -36 | -86 | 6 | L middle occipital lobe |
|  | 52 | 2.81 | 36 | -74 | 0 | R inferior occipital lobe |
| All participants H1/H1 > H2 carriers | 125 | 3.74 | -20 | -4 | -14 | L amygdala, L hippocampus |
|  | 671 | 3.33 | 12 | -28 | 30 | R middle cingulate |
|  | 50 | 3.04 | -46 | 38 | 24 | L middle frontal gyrus |
|  | 313 | 3.02 | 26 | 36 | 34 | R middle frontal gyrus |
|  | 61 | 2.87 | -16 | 48 | 40 | L superior frontal gyrus |
| Controls H1/H1 > H2 carriers | 1774 | 3.92 | 12 | -28 | 28 | R middle cingulate |
|  | 210 | 3.41 | -16 | -6 | -14 | L amygdala |
|  | 423 | 3.34 | 66 | -40 | 30 | R supramarginal gyrus |
|  | 183 | 3.29 | -60 | -44 | 32 | L supramarginal gyrus |
|  | 247 | 2.98 | 26 | 36 | 34 | R middle frontal gyrus |
|  | 104 | 2.83 | -40 | -12 | -2 | L insula |
|  | 125 | 2.82 | -18 | 58 | 26 | L superior frontal gyrus |
|  | 26 | 2.79 | -46 | 8 | -24 | L middle frontal gyrus |
|  | 27 | 2.74 | 26 | 6 | 16 | R putamen |

Peak activation coordinates are shown for each contrast, thresholded at p<0.01 (cluster extent >10 voxels) across the whole brain without correction for multiple comparisons. Values are derived from a 2 x 2 ANOVA in SPM with *MAPT* haplotype (H1 homozygotes vs. H2 carriers) and PD (patients vs. controls) as between subject factors and age and sex as covariates. Coordinates (X,Y,Z) refer to stereotactic space using the Montreal Neurological Institute (MNI) template. Anatomical labels were derived from the AAL atlas. PD, Parkinson’s Disease; L, left; R, right.

**^a^** there were no effects of H1/H1>H2 carriers across all participants on contrast 1 at this threshold

**^b^** there were no effects of H1/H1>H2 carriers in PD patients on contrast 2 at this threshold

References

Ashburner J. A fast diffeomorphic image registration algorithm. NeuroImage 2007; 38: 95–113.

Ashburner J, Friston KJ. Unified segmentation. NeuroImage 2005; 26: 839–851.

Ashburner J, Friston KJ. Computing average shaped tissue probability templates. NeuroImage 2009; 45: 333–341.

Barnes J, Ridgway GR, Bartlett J, Henley SMD, Lehmann M, Hobbs N, et al. Head size, age and gender adjustment in MRI studies: a necessary nuisance? NeuroImage 2010; 53: 1244–1255.

Brett M, Anton J-L, Valabregue R, Poline J-B. Region of interest analysis using an SPM toolbox [abstract]. Present. 8th Int. Conf. Funct. Mapp. Hum. Brain June 2-6 2002 Sendai Jpn. Available CD-ROM NeuroImage Vol 16 No 2 2002

Peelle JE, Cusack R, Henson RNA. Adjusting for global effects in voxel-based morphometry: gray matter decline in normal aging. NeuroImage 2012; 60: 1503–1516.

Tzourio-Mazoyer N, Landeau B, Papathanassiou D, Crivello F, Etard O, Delcroix N, et al. Automated anatomical labeling of activations in SPM using a macroscopic anatomical parcellation of the MNI MRI single-subject brain. NeuroImage 2002; 15: 273–289.
